# Bayesian Networks for Prescreening in Depression: Algorithm Development and Validation

**DOI:** 10.2196/52045

**Published:** 2024-07-04

**Authors:** Eduardo Maekawa, Eoin Martino Grua, Carina Akemi Nakamura, Marcia Scazufca, Ricardo Araya, Tim Peters, Pepijn van de Ven

**Affiliations:** 1 Department of Electronic and Computer Engineering University of Limerick Limerick Ireland; 2 Health Research Institute University of Limerick Limerick Ireland; 3 Departamento de Psiquiatria, Faculdade de Medicina da Universidade de Sao Paulo Universidade de Sao Paulo Sao Paulo Brazil; 4 Instituto de Psiquiatria, Hospital das Clinicas da Faculdade de Medicina da Universidade de Sao Paulo Faculdade de Medicina, Universidade de Sao Paulo Sao Paulo Brazil; 5 Centre for Global Mental Health King’s College London London United Kingdom; 6 Bristol Dental School University of Bristol Bristol United Kingdom

**Keywords:** Bayesian network, target depressive symptomatology, probabilistic machine learning, stochastic gradient descent, patient screening, depressive symptom, machine learning model, machine learning, survey, prediction, socioeconomic data sets, utilization, depression, mental health, digital mental health, artificial intelligence, AI, prediction, prediction modeling, patient, mood, anxiety, mood disorders, mood disorder, eHealth, mobile health, mHealth, telehealth

## Abstract

**Background:**

Identifying individuals with depressive symptomatology (DS) promptly and effectively is of paramount importance for providing timely treatment. Machine learning models have shown promise in this area; however, studies often fall short in demonstrating the practical benefits of using these models and fail to provide tangible real-world applications.

**Objective:**

This study aims to establish a novel methodology for identifying individuals likely to exhibit DS, identify the most influential features in a more explainable way via probabilistic measures, and propose tools that can be used in real-world applications.

**Methods:**

The study used 3 data sets: PROACTIVE, the Brazilian National Health Survey (Pesquisa Nacional de Saúde [PNS]) 2013, and PNS 2019, comprising sociodemographic and health-related features. A Bayesian network was used for feature selection. Selected features were then used to train machine learning models to predict DS, operationalized as a score of ≥10 on the 9-item Patient Health Questionnaire. The study also analyzed the impact of varying sensitivity rates on the reduction of screening interviews compared to a random approach.

**Results:**

The methodology allows the users to make an informed trade-off among sensitivity, specificity, and a reduction in the number of interviews. At the thresholds of 0.444, 0.412, and 0.472, determined by maximizing the Youden index, the models achieved sensitivities of 0.717, 0.741, and 0.718, and specificities of 0.644, 0.737, and 0.766 for PROACTIVE, PNS 2013, and PNS 2019, respectively. The area under the receiver operating characteristic curve was 0.736, 0.801, and 0.809 for these 3 data sets, respectively. For the PROACTIVE data set, the most influential features identified were postural balance, shortness of breath, and how old people feel they are. In the PNS 2013 data set, the features were the ability to do usual activities, chest pain, sleep problems, and chronic back problems. The PNS 2019 data set shared 3 of the most influential features with the PNS 2013 data set. However, the difference was the replacement of chronic back problems with verbal abuse. It is important to note that the features contained in the PNS data sets differ from those found in the PROACTIVE data set. An empirical analysis demonstrated that using the proposed model led to a potential reduction in screening interviews of up to 52% while maintaining a sensitivity of 0.80.

**Conclusions:**

This study developed a novel methodology for identifying individuals with DS, demonstrating the utility of using Bayesian networks to identify the most significant features. Moreover, this approach has the potential to substantially reduce the number of screening interviews while maintaining high sensitivity, thereby facilitating improved early identification and intervention strategies for individuals experiencing DS.

## Introduction

### Background

Improving the identification and management of depression in primary care remains a global challenge. A meta-analysis has revealed that in primary care, approximately 50% of patients with depression receive diagnoses, while around 15% acquire treatment [1]. While screening for depressive symptomatology (DS) holds significance, it alone falls short of being effective [2].

When evaluating individuals with DS, one approach involves the use of screening tools to determine who may require treatment and further investigation. Among these tools, the 9-item Patient Health Questionnaire (PHQ-9) is a widely used, self-administered questionnaire [3].

In certain scenarios, mental health information (such as the PHQ-9) may not be available, whereas other health or behavioral information that can be linked to an increased risk of depression may be abundant. Examples of such data are demographics, medical history, lifestyle indicators, and socioeconomic status. In such scenarios, it may be useful to leverage the available data to identify individuals likely to have DS such that these individuals can be targeted proactively for interventions. This proactive strategy has the potential to provide necessary support and care before symptoms escalate or result in severe consequences.

Data-driven approaches using machine learning offer an appealing opportunity to design better prescreening methodologies, particularly within primary care settings. Machine learning algorithms can identify patterns in the data that may not be obvious to human experts [4-6].

In many studies involving machine learning models, insights beyond the model development phase are not provided. The results usually provide a simple *Yes* or *No* for the presence of depression, usually operating as “black boxes” with an absence of transparency in the decision-making process [7,8]. There is a lack of understanding as to which features are most important in predicting DS.

There are instances where machine learning models offer some level of explainability [9-12]. However, the insights generated by these models often only consider the predictive power of the predictors. While this approach can help identify the most impactful predictor, it does not provide a probabilistic measure, which is essential for dealing with uncertainty. The use of probability-based measures in machine learning models can provide clinicians with a more nuanced understanding of an individual’s likelihood of experiencing depression.

Moreover, most machine learning models developed for detecting DS fail to provide applications on how to use the models effectively [13-16]. These studies do not develop practical tools to help users benefit from the machine learning models, focusing solely on the model development process. To encourage the use of machine learning models in clinical practice, it is essential to develop machine learning models that are designed with the end user in mind. This includes creating tools that can help practitioners effectively integrate the model into their practice.

The current literature on the use of machine learning models for predicting DS lacks a comprehensive integration of explainability and transparency to identify the important features associated with DS using only general health and socioeconomic data and in the absence of tools such as the PHQ-9 or any other depression-related features. In addition, there is a shortage of practical applications using machine learning to support clinical practice. In our proposed method, we aim to address these gaps.

### Objective

The first objective of this study is to establish a replicable prescreening proof-of-concept methodology for the detection of individuals with DS. Using solely general health and socioeconomic data, we aim to demonstrate the potential for such data in identifying individuals who might benefit from further screening. The second objective is to enable the identification of the most influential predictors with probabilistic insights into their importance, all based on the same general health and socioeconomic data. The third objective is to develop a tool that enables specialists to use the benefits of the methodology in their practice.

This paper’s structure comprises a *Methods* section detailing data preparation, the feature selection technique, and probabilistic insight extraction. It introduces a machine learning algorithm and its applications. The *Results* section presents cleaned data sets, chosen features, the most influential predictors, and the performance of the models and showcases the models’ utility in a practical scenario. The *Discussion* section concludes by summarizing findings, acknowledging limitations, and discussing implications.

## Methods

### Overview

Our proposed methodology for developing a machine learning model to assess people with DS was applied to 3 distinct Brazilian data sets. The first, known as the PROACTIVE data set [17], comprised individuals aged ≥60 years residing in socioeconomically deprived areas of Guarulhos city. The participants were registered in 20 primary care clinics in Guarulhos and were approached, according to a randomly ordered list, for a DS screening interview conducted either in person or via phone using a personalized app [18].

The other 2 data sets, Pesquisa Nacional de Saúde (PNS) 2013 and PNS 2019 [19], resulted from a Brazilian national health survey that assessed individuals aged ≥18 years in different sociodemographic groups and health behaviors. The surveys were conducted in 2013 and 2019 using a household approach where they applied stratified sampling.

All 3 studies used the PHQ-9, which is a 9-item questionnaire that serves as a screening tool for assessing DS. All questions are related to the previous 2 weeks, with responses to each question scored from 0 to 3, where 0 means “Not at all” and 3 means “Nearly every day.” The PHQ-9 cut-off score commonly used for DS is ≥10 [20-22], and we used this to create a binary classification target for the machine learning model. Summary statistics of the sociodemographic data and the prevalence of DS are presented in Tables S1, S2, and S3 in Multimedia Appendix 1 for the PROACTIVE, PNS 2013, and PNS 2019 data sets, respectively.

### Ethical Considerations

The PROACTIVE trial received approval from the Comitê de Ética em Pesquisa Faculdade de Medicina da Universidade de São Paulo and authorization from the Guarulhos Health Secretary (number 2.836.569). The Brazilian National Health Ethics Research Committee of the Brazilian National Health Council approved the PNS 2013 (number 328.159) and PNS 2019 (number 3.529.376) surveys. Anonymized versions of the PNS 2013 and PNS 2019 surveys are publicly available for download and analysis. All participants provided informed consent.

### Data Preparation

First, we randomly divided each data set into training (70%) and test (30%) sets. The development of the models is performed using the training data set with the test data set strictly used to test the performance of the created models on data not yet seen. Next, we created our response variable by summing the recorded responses of all the PHQ-9 score items for each participant and recording a 1 when the total sum was ≥10 and 0 otherwise. We only included participants who answered all 9 items, and the respondents were aged ≥18 years.

We dropped all features from the data sets related to depression, as our aim was to solely use data readily available in health platforms, such as general health and socioeconomic information. We also dropped features and patients with more than 20% missing values [23] and features with just 1 level in the responses obtained. Furthermore, we categorized all numeric features with more than 30 levels into 4 bins by quartiles (25, 50, 75, and 100).

We then transformed all features into ordinal numbers. To address any remaining missing values, we adopted an approach discussed in the study by Enders [24] by creating a new missing class and assigning it a value of 0. This choice was based on the fact that not all features had a value of 0 and that 0 is a value close to the range of the feature values. This also facilitates model development, as the standardization process that we applied after the missing imputation step is less sensitive when the range values are close to each other. Standardization is applied to ensure that all features have a similar scale, which helps in comparing the importance of different features and improving the model’s overall performance [25].

### Feature Selection

The development of a machine learning model generally involves selecting relevant features to be used as inputs. A total of 1 approach to this task is constructing a Bayesian network (BN), which is a directed acyclic graph composed of nodes and edges. In this context, nodes represent the features and the directed edges represented by arrows illustrate the relationships among them [26].

BNs have been successfully applied in different scenarios, such as feature selection [27-29], model prediction [30-32], and providing insights for decision-making [33-36]. Moreover, a BN is a useful tool for visualizing and interpreting complex relationships between features. This enables the identification of critical features and their impact on the outcome.

BNs can be constructed using two primary methods, namely (1) manual node definition and edge direction [37] or (2) learning through data [38,39].

In our approach, we apply the Incremental Association Markov Blanket algorithm to learn the BN from data [40]. The Markov blanket (MB) of a node X, represented by MB(X), is characterized by its parents (nodes that have arrows pointing toward X), children (nodes that receive arrows from X), and spouses (nodes with arrows leading to children of X, yet not linked to X).

To evaluate the confidence level of the BN learned from the data, we used a bootstrap approach [41] by generating 1000 samples of the BN. We then computed the probabilities of having an edge between every pair of nodes (Xi, Xj; known as strength) and the probabilities of having a directed edge from Xi to Xj and from Xj to Xi (known as direction) [42]. To construct our final BN, we selected the edges with a strength of 50% and higher [43] and set their direction to correspond with the majority of the bootstrap models.

Subsequently, we conducted another test to assess the strength of the BN by performing an independence test between every pair of nodes using mutual information [44]. We used an independence test because it can investigate whether 2 features are statistically independent, implying that the occurrence or value of 1 feature does not influence the other. If the test yielded insufficient evidence of nonindependence (*P*≥.05), then that edge was removed from the network.

Conventionally, in the literature [45,46], BN-based feature selection is predominantly performed using the MB of the outcome of interest. However, we also tested the efficacy of using all the features connected to the outcome by a path (we call this All-path-features). A feature was considered part of this path if it was possible to reach the outcome node by traversing the edges and nodes in between, regardless of the directions of the edges. Therefore, we tested 2 scenarios of the model: 1 using All-path-features in the path and another using only the MB of the outcome.

### BN Parameter Learning

Once the BN structure is constructed, it is possible to assess the relationships between the features. These relationships are probabilistically expressed through conditional probability distributions (CPDs) [47]. The process of estimating the CPD is known as parameter learning. The CPD specifies the probability distribution for each node given its parents. To estimate the CPD, we used the Bayesian method [48]. The Bayesian method is a powerful tool for estimating the CPD, as it takes into account both prior knowledge and observed data.

In this study, we computed the CPD table of the outcome node given its parents. The parents represent the features on which our outcome is conditionally dependent, and the CPD table provides the probability values of having DS for different combinations of parent values. We examined the combinations of parent values that exhibited the most discriminatory power when considering individuals with DS.

The data preparation, construction, and parameter learning of the BN were implemented using R 4.2.2 with libraries bnlearn, parallel, and base. R is freely available open source software (R Foundation for Statistical Computing).

### Training of Prediction Models

In the training phase of model development, particularly in a classification problem with 2 classes, it is common practice to oversample the minority class [49-51]. This is because by oversampling the minority class, we can improve the model’s ability to learn from both classes and achieve better overall performance.

For the development of the models, our approach applied stochastic gradient descent (SGD), which is a widely used optimization algorithm in machine learning. The objective of SGD is to minimize an error function through an iterative process where the model’s parameters are updated at each step until the algorithm converges. SGD is known for its scalability [52], stability, and robustness [53] and has shown good performance across different domains [54-56].

One of the drawbacks of using SGD is that finding the optimal combination of hyperparameters for each data set can be challenging. We used BayesSearchCV from Python’s skopt package, which applies both Bayesian optimization and 5-fold cross-validation to evaluate each model’s performance [57]. The selection of the optimal hyperparameter set for each data set was done by selecting the configuration that yielded the highest area under the receiver operating characteristic curve (AUC-ROC) metric.

The study focused on tuning 3 hyperparameters: the loss function, penalty term, and α coefficient. The loss function characterizes the relationship between the model’s predictions and the actual values. A total of 3 types of loss functions were examined: Hinge, Modified Huber, and Log [58,59].

The penalty term corresponds to a regularization technique aimed at enhancing the model’s generalization capability. In addition, the α coefficient, a positive value, controls the level of regularization applied. In this study, 3 types of regularization for the penalty term were considered: L1 [60], L2 [61], and elastic net [62].

To explore the effect of the α coefficient on regularization, a range of values from 0.000001 to 1,000,000 were used.

### Evaluation of the Models

We evaluated the models using two scenarios: (1) All-path-features and (2) the MB of the outcome, as mentioned in the *Feature Selection* section. For each scenario, we used the best set of hyperparameters discussed in the *Training of Prediction Models* section.

To validate each model, we once again applied 5-fold cross-validation on top of the cross-validation for hyperparameter tuning explained in the previous section. For each fold, we recorded the threshold that optimized both sensitivity and specificity simultaneously determined by the Youden index. Afterward, by averaging the thresholds obtained from the folds, we analyzed the metrics of AUC-ROC, sensitivity, and specificity. In addition, we calculated the mean and SD of these metrics across the folds. Sensitivity, specificity, and AUC-ROC were chosen as evaluation metrics to assess the performance of the models, given their advantages in the screening process.

To assess the performance of each machine learning model, we used the test data, which was set aside during the data preparation stage. We used sensitivity, specificity, and AUC-ROC to assess the performance of the model, applying their respective thresholds calculated according to the previous paragraph. Then, we compared the results to those obtained from the training data. By comparing these results, we gained insights into the model’s generalization capabilities and its performance on new and unseen instances.

The model development was implemented in Python 3.7.7 using packages SGDClassifier, CalibratedClassifierCV from sklearn, and BayesSearchCV from skopt. Python is freely available open source software (Python Software Foundation).

### Application of the Models

Having identified the most important features and created a model to use these features to predict DS for each data set, the next step was to analyze how the models can help target people with DS and what benefits they offer. To this end, we used the test data, which were previously only used to evaluate the performance of the models, to illustrate the relationship between the reduced screening interviews that can be obtained by selecting screening participants based on the developed model and the sensitivity and specificity of the overall screening methodology.

To illustrate the benefits of using these models for selecting screening participants, consider a specific cohort with a DS prevalence of 10%. If we were to screen this cohort randomly, in a study with 100 participants having DS, 1000 individuals would need to be screened. From a public health perspective, 10 individuals would need to be screened for every identified individual.

**Figure 1:**
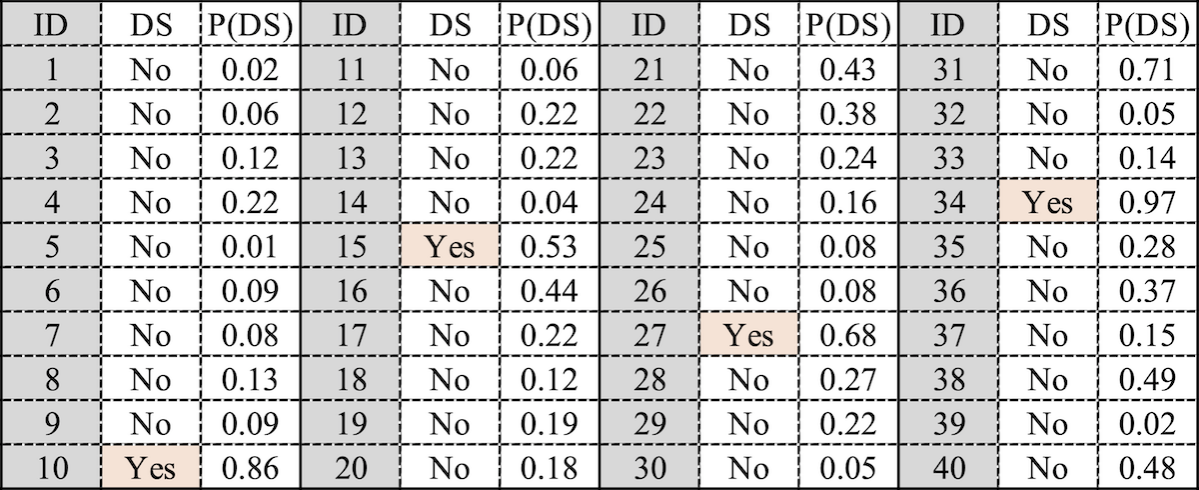
Example of a random screening list. DS: depressive symptomatology; P(DS): probability of having depressive symptomatology.

To assess the effectiveness of the models, consider a scenario where we need to screen 40 individuals using a random approach (Figure 1). For a 10% DS prevalence, we would identify 4 people having DS.

The models developed by our approach can be used to prioritize the individuals with the highest risk of DS for screening. This is because the model provides a probability score for each individual having DS. Hence, by ordering the cohort by this probability, and screening individuals in order of decreasing the probability of DS, the screening process is expected to be much more efficient.

**Figure 2:**
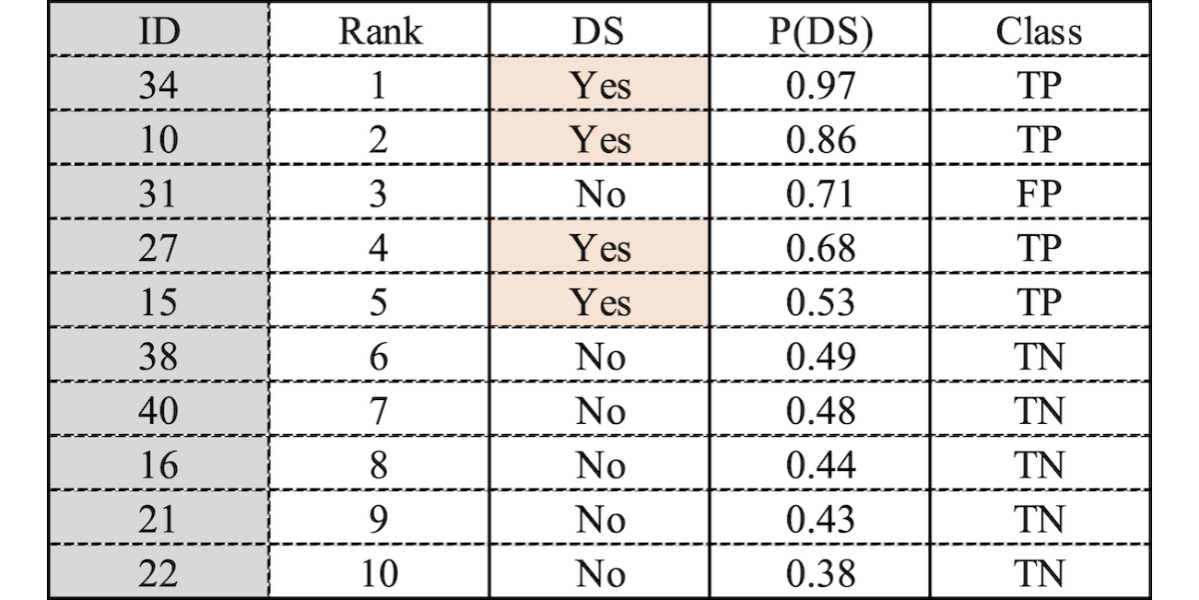
Example of a ranked list provided by the models. Class=comparing predictions with observed values. DS: depressive symptomatology; FP: false positive; P(DS): probability of having depressive symptomatology; TN: true negative; TP: true positive.

A ranked list example is illustrated in Figure 2. If we use our models and start to screen individuals from 1 to 10, after screening 5 individuals (based on the probability score of having DS given by our models with a threshold >0.5), we would expect to identify 4 true positives (TPs) who have DS and 1 false positive (FP) who does not have DS. To identify 4 individuals with DS using our models, it would be necessary to screen a total of 5 people. Therefore, by using our models and screening only 5 people, we achieve the same outcome as screening 40 people randomly, representing a reduction of 87.5% in screening interviews.

We can measure the reduction in number of screening interviews. This value will provide us with a clear understanding of how much the model can save compared with the random screening approach and is illustrated in equation 1:

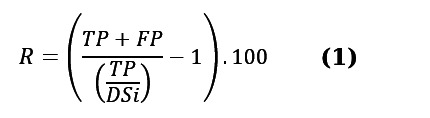



Where *R* is the reduction in the number of screening interviews in %, *TP* is the number of TP cases, *FP* is the number of FP cases, and *DSi* is the prevalence rate of DS.

Another crucial aspect of the developed models is their capability to simulate the relationship between sensitivity and the reduction in screening efforts. For example, suppose a practitioner believes that the achieved sensitivity using the default threshold of 0.5 is insufficient, it is possible to fine-tune the sensitivity by selecting a new threshold. By doing so, the models can calculate the corresponding reduction in screening interviews associated with the adjusted sensitivity value.

## Results

### Data Preparation

After preprocessing the training data as discussed in the previous section, we were left with 12 out of 33 features and 3854 out of 3961 observations for the PROACTIVE data set. For PNS 2013, we were left with 218 features and 77,376 observations out of the initial 1000 and 155,432, respectively. Similarly, for PNS 2019, we had 254 out of 1087 features and 111,548 out of 205,434 observations.

Despite PNS 2019 having an additional 87 features compared with PNS 2013, the features present in PNS 2013 are also included in PNS 2019. The divergence lies in the 2019 survey, wherein certain questions were broken down into multiple components. On the other hand, PROACTIVE incorporates distinct features from both PNS 2013 and 2019 data sets.

### Feature Selection

We built the BN from data using 1000 bootstrapped samples. We then performed the additional independence test on all the edges as described in the *Feature Selection* section. For the PROACTIVE data set, the connection from HYPERTENSION to AGE was dropped (*P*≥.05). For PNS 2013 and PNS 2019, no edge had to be removed. The BNs for PROACTIVE, PNS 2013, and PNS 2019, with 12, 19, and 29 features, respectively, are displayed in Figures S1-S3 in Multimedia Appendix 1.

For the PROACTIVE data set, the MB of the outcome node consisted of postural balance problems, shortness of breath, and how old people feel they are. In the PNS 2013 data set, the MB nodes were related to the ability to do usual activities, chest pain, chronic back problems, and sleep problems. Finally, in the PNS 2019 data set, the MB nodes were related to the ability to do usual activities, chest pain, verbal abuse, and sleep problems. Detailed descriptions of these features can be found in Tables S4, S5, and S6 of Multimedia Appendix 1 for PROACTIVE, PNS 2013, and PNS 2019, respectively.

### BN Parameter Learning

For each data set, we analyzed the probability of having DS given their MB nodes. As detailed in Table 1 for PROACTIVE, the features of postural balance and shortness of breath each have 2 levels (“Yes” or “No”), and the feature of how old people feel they are has 4 levels, with the minimum being up to 50 years and the maximum being >70 years. In Table 1, we present the probabilities of DS for those not having a postural balance problem, not having a shortness of breath issue, and feeling up to 50 years old, against having both issues and feeling more than 70 years old. All individuals in this data set are aged ≥60 years.

**Table 1:** Probability of having depressive symptomatology in PROACTIVE for 2 example scenarios.

Postural balance^a^	How old people feel they are?^b^	Shortness of breath^c^	P(DS)^d^
No	Up to 50 years	No	0.08
Yes	>70 years	Yes	0.75

^a^Do you have problems with postural balance?

^b^In general (or most of the time), how old do you feel?

^c^Have you ever experienced shortness of breath while walking, climbing stairs, or with changes in temperature (eg, when it is hot or cold)?

^d^P(DS): probability of having depressive symptomatology.

For the PNS 2013 and PNS 2019 data sets, the MB features are binary, with 2 possible values: “Yes” or “No.” Thus, Tables 2 and 3 present 2 distinct scenarios, one where all features have a “Yes” value, and the other where all features have a “No” value.

**Table 2:** Probability of having depressive symptomatology in Pesquisa Nacional de Saúde 2013 for 2 example scenarios.

Ability to do usual activities^a^	Chest pain^b^	Chronic back problems^c^	Sleep problems^d^	P(DS)^e^
Yes	Yes	Yes	Yes	0.66
No	No	No	No	0.03

^a^In the past 2 weeks, have you been unable to perform any of your usual activities (such as work, school, playing, and household chores) due to health reasons?

^b^Do you feel chest pain or chest discomfort when walking on a hill, going up one flight of stairs, or fast walking?

^c^Do you have any chronic spinal problems such as chronic back or neck pain, lumbago, sciatica, vertebral, or disc problems?

^d^In the past 2 weeks, have you used any medication to help you sleep?

^e^P(DS): probability of having depressive symptomatology.

**Table 3:** Probability of having depressive symptomatology in Pesquisa Nacional de Saúde 2019 for 2 example scenarios.

Ability to do usual activities^a^	Chest pain^b^	Verbal abuse^c^	Sleep problems^d^	P(DS)^e^
Yes	Yes	Yes	Yes	0.79
No	No	No	No	0.04

^a^In the past 2 weeks, have you been unable to perform any of your usual activities (such as work, school, playing, and household chores) due to health reasons?

^b^Do you feel chest pain or chest discomfort when walking on a hill, going up one flight of stairs, or fast walking?

^c^In the past 12 months, has anyone yelled or cursed you?

^d^In the past 2 weeks, have you used any medication to help you sleep?

^e^P(DS): Probability of having depressive symptomatology.

The results presented in Table 1 for the PROACTIVE data set indicate that individuals who report postural balance problems, episodes of shortness of breath, and feeling >70 years old have a higher probability of having DS. Specifically, if a person reports all 3 of these symptoms, the probability of having DS is 0.75, which is over 8 times higher than if the person reports feeling <50 years old and having no issues with postural balance or being out of breath.

In both PNS 2013 and PNS 2019, 3 features remained consistent over the 6-year period in both data sets (ability to do usual activities, chest pain, and sleep problems). The only difference is that in PNS 2013, feature chronic back problems as shown in Table 2 is replaced by those related to verbal abuse as illustrated in Table 3. The results reveal that answering “Yes” to all 4 questions results in a probability of having DS of 0.66 for PNS 2013 and 0.79 for PNS 2019. On the other hand, if a person answers “No” to all 4 questions, the probability of having DS is only 0.03 and 0.04, respectively.

### Training of Prediction Models

The results obtained from the training process of our models are presented in Tables 4-6 for the 3 data sets. These tables provide the sensitivity and specificity determined by the thresholds of 0.444, 0.412, and 0.472 for PROACTIVE, PNS 2013, and PNS 2019, respectively. Furthermore, they present the AUC-ROC metrics for two scenarios: (1) using All-path features of the BN and (2) using the MB of the outcome.

Each of the 3 tables includes sensitivity, specificity, and AUC-ROC values for 5 folds, which were obtained using a 5-fold cross-validation approach with the best set of hyperparameters, as described in the *Training of Prediction Models* section. These metrics specifically correspond to the validation sets used during the cross-validation process.

Upon analyzing the results across all 3 data sets, we observed consistent outcomes. The mean values of sensitivity, specificity, and AUC-ROC for each fold closely align with the individual values, and the SD is low.

In PROACTIVE (Table 4), the optimal parameters were determined to be α=.200, loss function=modified_huber, and penalty term=L2 for the All-path-features scenario. The MB scenario required a different set of parameters, specifically α=.0005, loss function=log, and penalty term=elastic net.

For PNS 2013 (Table 5), the optimal hyperparameters for model tuning were found to be an α value of .003, hinge loss function, and L2 penalty term for the all-features scenario. The MB scenario yielded an α value of .006, log loss function, and L1 penalty term.

The best parameters for the PNS 2019 (Table 6) model were α=.003, loss function=log, and penalty term=elastic net. The parameters for the MB scenario were α=.083, loss function=log, and penalty term=L2.

**Table 4:** PROACTIVE—sensitivity, specificity, and area under the receiver operating characteristic curve (AUC-ROC) values in 5-fold cross-validation.

Metrics		F1^a^	F2^b^	F3^c^	F4^d^	F5^e^	Values, mean (SD)
**All-path-features**							
	Sensitivity	0.813	0.751	0.764	0.764	0.748	0.768 (0.024)
	Specificity	0.640	0.598	0.634	0.642	0.647	0.632 (0.018)
	AUC-ROC	0.795	0.723	0.767	0.766	0.765	0.763 (0.026)
**Markov blanket**							
	Sensitivity	0.678	0.673	0.637	0.699	0.621	0.662 (0.029)
	Specificity	0.725	0.689	0.748	0.691	0.761	0.723 (0.029)
	AUC-ROC	0.771	0.713	0.758	0.758	0.759	0.751 (0.022)

^a^F1: fold 1 of cross-validation.

^b^F2: fold 2 of cross-validation.

^c^F3: fold 3 of cross-validation.

^d^F4: fold 4 of cross-validation.

^e^F5: fold 5 of cross-validation.

**Table 5:** Pesquisa Nacional de Saúde 2013—sensitivity, specificity, and area under the receiver operating characteristic curve (AUC-ROC) values in 5-fold cross-validation.

Metrics			F1^a^		F2^b^		F3^c^		F4^d^		F5^e^		Values, mean (SD)
**All-path-features**													
	Sensitivity	0.731		0.738		0.743		0.733		0.736		0.736 (0.004)	
	Specificity	0.744		0.732		0.738		0.728		0.736		0.735 (0.005)	
	AUC-ROC	0.807		0.805		0.809		0.798		0.805		0.805 (0.004)	
**Markov blanket**													
	Sensitivity	0.712		0.726		0.722		0.717		0.720		0.756 (0.003)	
	Specificity	0.716		0.703		0.714		0.697		0.709		0.708 (0.007)	
	AUC-ROC	0.758		0.758		0.759		0.750		0.756		0.756 (0.004)	

^a^F1: fold 1 of cross-validation.

^b^F2: fold 2 of cross-validation.

^c^F3: fold 3 of cross-validation.

^d^F4: fold 4 of cross-validation.

^e^F5: fold 5 of cross-validation.

**Table 6:** Pesquisa Nacional de Saúde 2019—sensitivity, specificity, and area under the receiver operating characteristic curve (AUC-ROC) values in 5-fold cross-validation.

Metrics			F1^a^		F2^b^		F3^c^		F4^d^		F5^e^		Values, mean (SD)
**All-path-features**													
	Sensitivity	0.704		0.712		0.703		0.710		0.717		0.709 (0.005)	
	Specificity	0.763		0.760		0.759		0.757		0.761		0.762 (0.002)	
	AUC-ROC	0.80		0.804		0.80		0.804		0.810		0.804 (0.004)	
**Markov blanket**													
	Sensitivity	0.744		0.751		0.647		0.750		0.655		0.709 (0.047)	
	Specificity	0.707		0.704		0.773		0.710		0.771		0.733 (0.032)	
	AUC-ROC	0.765		0.767		0.766		0.769		0.772		0.768 (0.003)	

^a^F1: fold 1 of cross-validation.

^b^F2: fold 2 of cross-validation.

^c^F3: fold 3 of cross-validation.

^d^F4: fold 4 of cross-validation.

^e^F5: fold 5 of cross-validation.

### Evaluation of the Models

The evaluation results of the models on the test data are presented in Table 7. In addition, we provide the metrics obtained from analyzing the entire training data and the mean of the 5-fold cross-validation. It is important to note that the whole data evaluation approach differs from the 5-fold cross-validation method. Instead of comparing the average metrics across the 5 folds in the validation set as if they were separate models, we assess the overall performance on the complete training data set.

**Table 7:** Sensitivity, specificity, and area under the receiver operating characteristic curve on test data using All-path-features.

			PROACTIVE		Pesquisa Nacional de Saúde 2013		Pesquisa Nacional de Saúde 2019
**Sensitivity**							
	Training	0.766		0.735		0.708	
	Cross-validation	0.768		0.736		0.709	
	Test	0.717		0.741		0.718	
**Specificity**							
	Training	0.633		0.737		0.762	
	Cross-validation	0.632		0.735		0.762	
	Test	0.644		0.737		0.766	
**Area under the receiver operating characteristic curve**							
	Training	0.765		0.806		0.804	
	Cross-validation	0.763		0.805		0.804	
	Test	0.736		0.801		0.809	

The sensitivity specificity and AUC-ROC metrics obtained from the whole data evaluation, illustrated in Table 7, exhibit consistency with those obtained through the 5-fold cross-validation approach. Moreover, these metrics are not considerably different from the sensitivity, specificity, and AUC-ROC observed in the test data.

**Figure 3:**
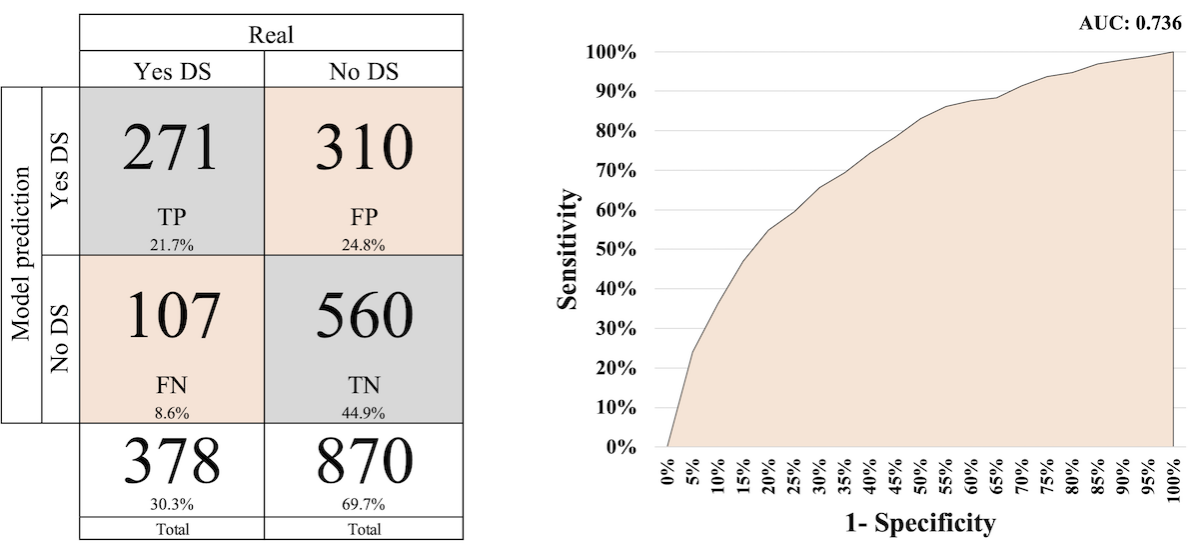
Confusion matrix and area under the receiver operating characteristic curve for PROACTIVE. DS: depressive symptomatology; FN: false negative; FP: false positive; TN: true negative; TP: true positive.

**Figure 4:**
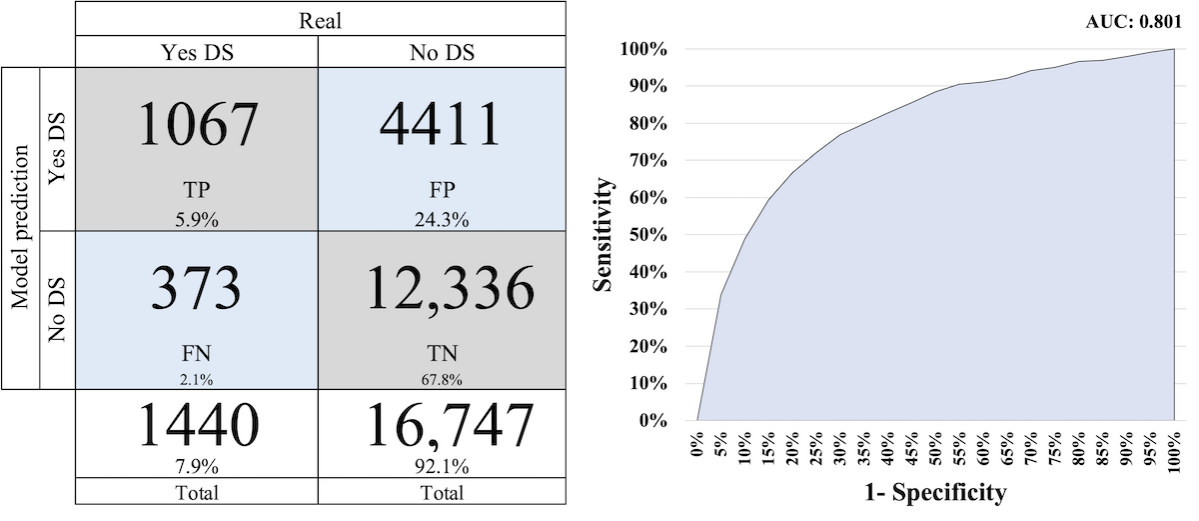
Confusion matrix and area under the receiver operating characteristic curve for Pesquisa Nacional de Saúde 2013. DS: depressive symptomatology; FN: false negative; FP: false positive; TN: true negative; TP: true positive.

**Figure 5:**
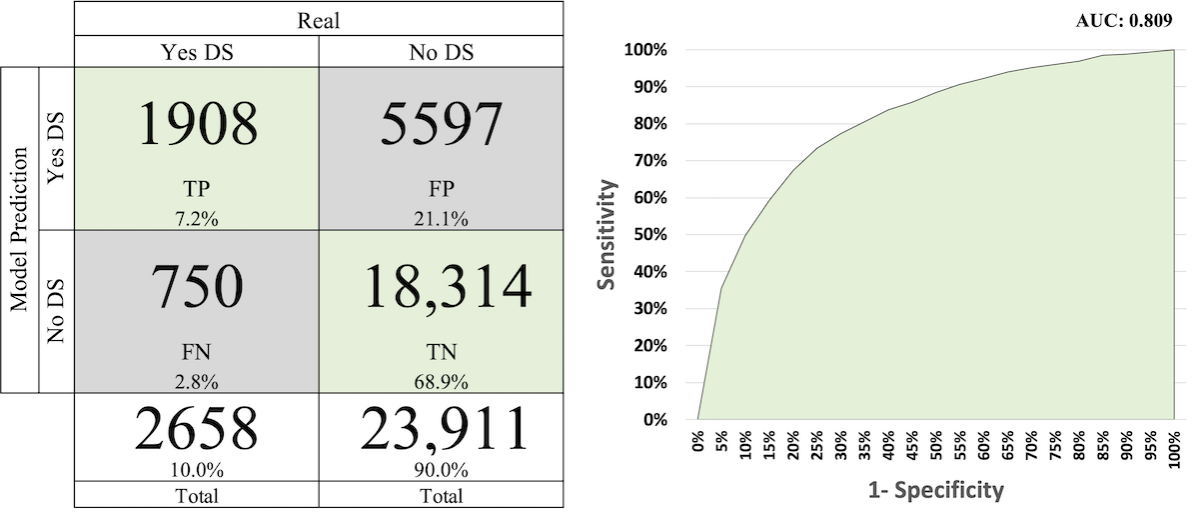
Confusion matrix and area under the receiver operating characteristic curve for Pesquisa Nacional de Saúde 2019. DS: depressive symptomatology; FN: false negative; FP: false positive; TN: true negative; TP: true positive.

Figures 3-5 display the AUC-ROC graph and confusion matrices for PROACTIVE, PNS 2013, and PNS 2019, respectively. The confusion matrix is based on the threshold of each model applied to the test data.

In Figure 3, the confusion matrix reveals that out of a total of 581 cases above the threshold (TPs+FPs), 271 cases actually have DS. In addition, there are 107 cases below the threshold that have DS (false negatives). Moreover, the model for the PROACTIVE data set accurately classifies 831 cases (66.6%: TPs+true negatives) out of a total of 1248 cases.

Comparing Figures 4 and 5 with Figure 3, we observe better results. Both figures exhibit higher AUC-ROC values, with 0.801 for PNS 2013 and 0.809 for PNS 2019. In terms of correctly classifying cases, the models achieved a success rate of 73.7% for PNS 2013 and 76.1% for PNS 2019.

### Application of the Models

Our approach offers a high degree of flexibility in terms of adjusting the threshold to suit a desired sensitivity value. To achieve this, we can leverage the test data to simulate the trade-off between sensitivity and screening interviews reduction using equation 1 outlined in the *Methods* section.

The prevalence of DS in the PROACTIVE, PNS 2013, and PNS 2019 data sets is 30.3%, 7.9%, and 10% as illustrated in Figure 3, Figure 4, and Figure 5, respectively. The prevalence of DS in the PROACTIVE data set is more than 3 times that in the PNS data sets. Hence, it is reasonable to expect that both PNS models will exhibit superior performance with respect to screening interviews reduction in comparison to PROACTIVE. This is because when using a random screening approach, a higher prevalence of DS within a particular cohort leads to more efficient identification of affected individuals, as opposed to a cohort with a lower prevalence.

**Figure 6:**
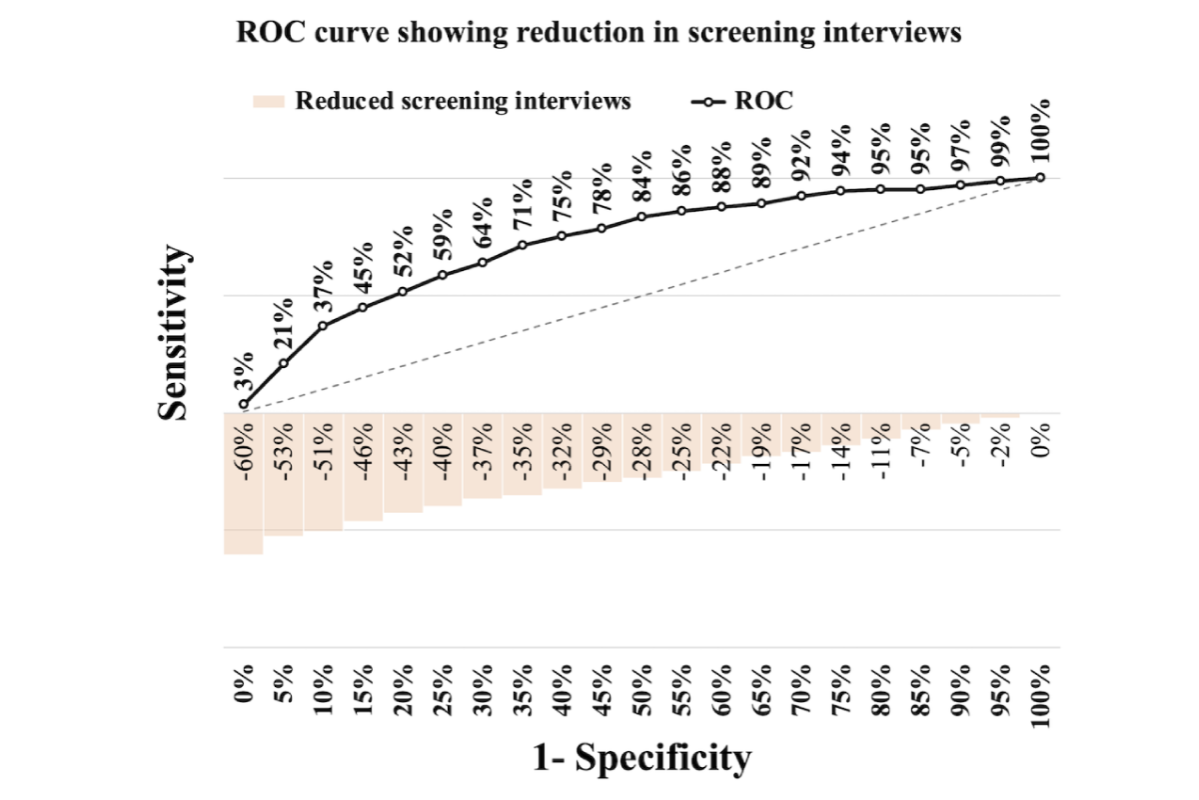
PROACTIVE—trade-off between sensitivity versus reducing screening interviews. ROC: receiver operating characteristic.

**Figure 7:**
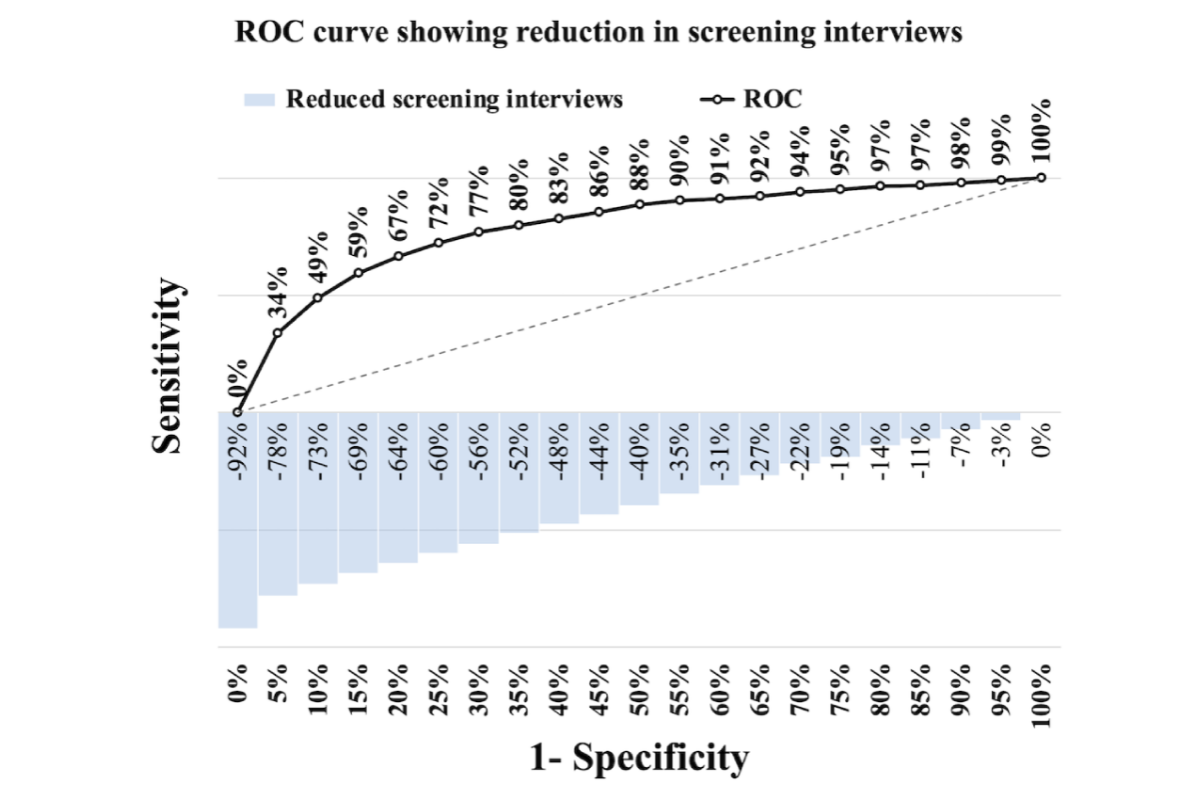
Pesquisa Nacional de Saúde 2013—trade-off between sensitivity versus reducing screening interviews. ROC: receiver operating characteristic.

**Figure 8:**
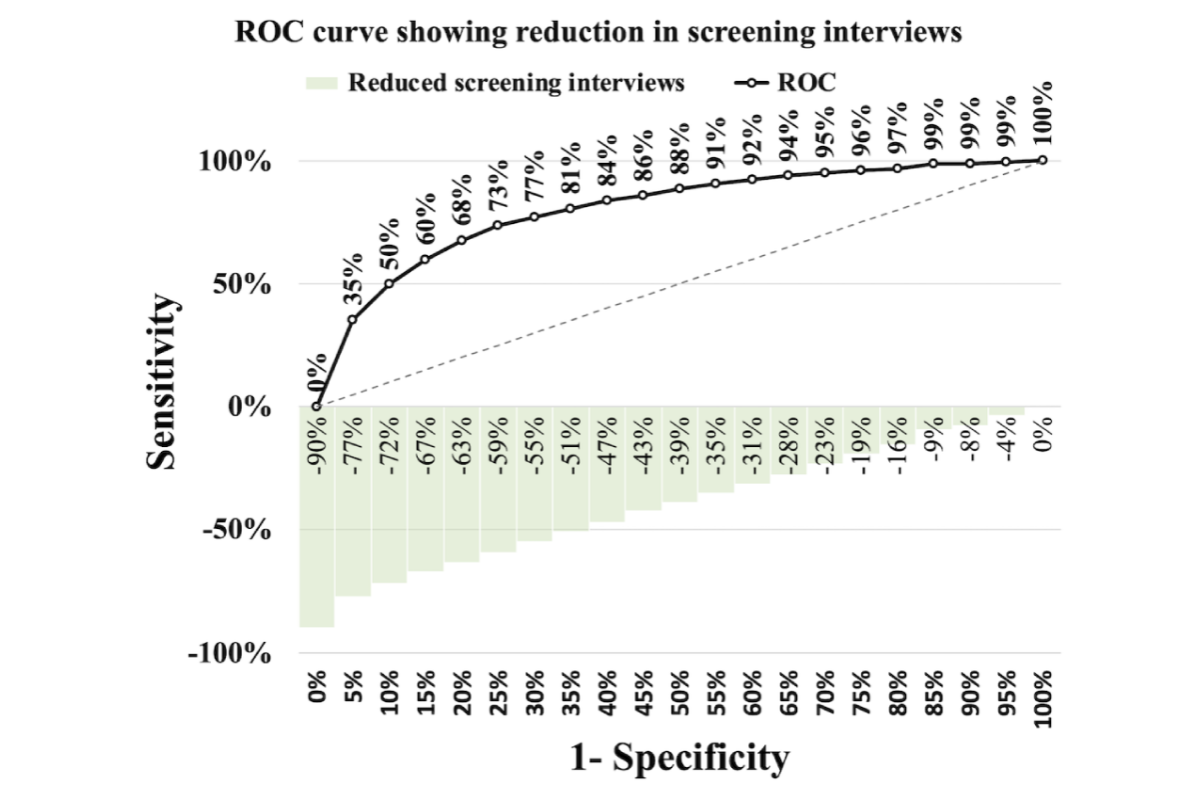
Pesquisa Nacional de Saúde 2019—trade-off between sensitivity versus reducing screening interviews. ROC: receiver operating characteristic.

Figures 6-8 illustrate the receiver operating characteristic curves, where the bars on the x-axis represent the reduction in screening interviews as a percentage. These graphs demonstrate the trade-off between the sensitivity and the reduction in screening interviews achieved by using our models.

As an example, according to Table 7, the calculated threshold in the PROACTIVE data set corresponds to a sensitivity of 0.717. In Figure 6, this sensitivity reduces screening interviews by 35%. Furthermore, even when aiming for higher sensitivities, such as 78% or 92%, using the model developed for this data set can still result in reducing interviews by 29% or 17%, respectively.

The key finding is that for any sensitivity above 0.640, using this model consistently outperforms a normal random approach (the screening interviews reduction is higher than 1-sensitivity). This holds even with the high prevalence of DS in this cohort, as demonstrated by Figure 6.

In the case of both PNS models, it is apparent from Figures 7 and 8 that they outperform the PROACTIVE model in Figure 6. Although both models exhibit considerable improvements, the PNS 2013 model demonstrates a slightly better performance than the PNS 2019 model. Specifically, the results indicate that at a sensitivity of 88%, the PNS 2013 model achieves a 40% reduction in screening interviews, while the PNS 2019 model achieves a 39% reduction. The reduction at the same sensitivity level of 88% achieved by the PROACTIVE model is 22%. Furthermore, for both PNS data sets, using our models is always superior to using a random approach.

## Discussion

### Overview

To enhance the development of our models, we conducted a comparison between 2 scenarios regarding feature selection. In the first scenario, we used All-path-features nodes from our BN, while in the second scenario, we focused on using only the MB of the outcome. By analyzing and contrasting these 2 scenarios, we aim to gain a comprehensive understanding of the impact that including or excluding these specific nodes has on our model.

The results of our analysis consistently showed that using All-path-features from the BN yielded superior outcomes compared with using only the MB of the outcome. Furthermore, we used the test data in the All-path-features scenario to evaluate the performance of the models for each data set. This approach ensures that the model’s performance is assessed in a fair manner using previously unseen data, reflecting its real-world performance.

Using the optimal threshold that maximizes both sensitivity and specificity, determined by the Youden index, can be beneficial. This approach ensures more reliable and accurate diagnoses by minimizing the errors of missing a positive case (false negatives) or incorrectly diagnosing a negative case as positive (FPs).

The PNS 2013 and PNS 2019 data sets stand as valuable pillars of Brazil’s national health data landscape. The models have consistently highlighted 3 features—the ability to do usual activities, chest pain, and sleep problems. This result across a 6-year span underscores these attributes’ robustness as good indicators for detecting DS, thereby offering valuable insights for health care services. However, it is important to reapply the methodology as new data sets emerge to check for patterns and new insights.

Our assessment revealed that both the models trained on the PNS 2013 and PNS 2019 data sets outperformed the PROACTIVE data set model. This can be attributed to the larger sample sizes and lower prevalence rates of DS in the PNS data sets. It is worth noting that the PROACTIVE study exclusively concentrated on individuals aged ≥60 years. Larger data sets provide a more diverse representation of the population, leading to improved generalization of the model’s predictions. In addition, the lower prevalence of DS in these data sets implies that a random screening approach would require screening a larger number of individuals to identify those with DS. In this context, the model’s ability to target individuals with a higher probability of having DS can markedly reduce screening interviews.

Despite our emphasis on developing a methodology using general health and socioeconomic data without relying on features related to depression, our approach outperformed studies using similar nondepression-related features. For instance, a study conducted with university undergraduates in Bangladesh used machine learning models to predict depression. The predictors included basic information such as academic year and cumulative grade point average, without using the PHQ-9 or any depression-related information. The models achieved an AUC-ROC ranging from 0.694 to 0.802, sensitivity ranging from 0.32 to 0.53, and specificity ranging from 0.86 to 0.87 [63]. Another study, conducted in Japan, used machine learning to predict depressive symptoms using sociodemographic and biological metabolite information, also without relying on the PHQ-9 or questionnaires assessing depressive symptoms. Their models achieved an AUC-ROC ranging from 0.53 to 0.68 but did not report results for sensitivity and specificity [64].

### Principal Findings

Our models exhibit almost the same sensitivity, specificity, and AUC-ROC values when applied to both the training and test data across all 3 data sets. This finding suggests that our approach is capable of generalizing effectively to unseen data, demonstrating its robustness and reliability.

It is worth noting that even when using only the MB, the results obtained were still satisfactory, making it a viable option for reducing the number of questions required to predict DS. Furthermore, the probabilistic measures extracted from the MB of the outcome demonstrated strong discriminatory power. This emphasizes that the features within the MB hold the utmost importance in the model across all 3 data sets.

The output of the models offers a visual representation of the relationship between sensitivities and the associated reduction in screening interviews. This gives health care providers the flexibility to adjust the desired sensitivity of the models according to their specific requirements. Such adaptability enhances the utility of the model as a proof-of-concept in clinical settings, making it particularly beneficial in environments with limited resources.

### Limitations

While a PHQ-9 score of 10 or higher can serve as a useful indicator for DS, it should be interpreted in conjunction with other clinical information and within the context of an individual’s unique circumstances. If available in the data sets, it would be straightforward with our methodology to predict different outcomes beyond PHQ-9 scores. However, the methodology may require some adjustments in the algorithm.

While our proposed methodology demonstrated good performance in all 3 data sets, it is important to note that there is a possibility of the model not performing well on certain data sets. This could happen if the features in the data set do not have a strong correlation with the outcome or if the prevalence of DS in a specific cohort is high.

The consistent prominence of the same 3 essential features in both PNS 2013 and PNS 2019 is noteworthy, but this information holds relevance primarily within Brazilian primary care settings. Across diverse cultures, different features might emerge as the most important indicators. Furthermore, it is important to consider the potential for these features to dynamically shift in significance over time, depending on the readily available data.

We acknowledge that having access to the readily available data may require obtaining permissions. Despite the data being accessible, various legal and ethical considerations may necessitate obtaining explicit permissions or approvals before its use.

Although the use of BN for feature selection yielded consistent results, it is worth noting that constructing the model using bootstrap can be very hardware demanding. In addition, the feasibility of this approach depends on the size of the data set in terms of the number of features, the range of values of the features, and the number of observations.

### Conclusions

We presented a data-driven proof-of-concept methodology for identifying individuals with DS using alternative features beyond common mental health screening questionnaires. This approach was tested on 3 distinct data sets, yielding consistent results that indicate a strong generalization of the methodology for targeting individuals who could benefit from further screening.

Using BNs, we were able to identify the most influential features (ie, the MB of the outcome) and extract insights using probabilistic measures through parameter learning. Our analysis revealed that for the PROACTIVE data set, a study that screened Brazilians aged ≥60 years, the most influential features were related to postural balance, shortness of breath, and how old people feel they are. For the PNS data sets, which screened a national sample of Brazilians aged ≥18 years, the influential features of the 2013 data set were related to the ability to do usual activities, chest pain, chronic back problems, and sleep problems. In the PNS 2019 data set, the same features were found to be important as in PNS 2013, except for chronic back problems, which were replaced by a feature related to verbal abuse.

Finally, it has been demonstrated through empirical analysis that using the proposed models results in a considerable reduction of the screening interviews (up to 52%) while maintaining a sensitivity of 80%.

## Appendix

Multimedia Appendix 1 Supplementary tables and figures.

## Abbreviations

AUC-ROC: area under the receiver operating characteristic curve
BN: Bayesian network
CPD: conditional probability distribution
DS: depressive symptomatology
FP: false positive
MB: Markov blanket
PHQ-9: 9-item Patient Health Questionnaire
PNS: Pesquisa Nacional de Saúde
SGD: stochastic gradient descent
TP: true positive
