# Supplementary material for: Bayesian Networks for Prescreening in Depression: Algorithm Development and Validation
Source: JMIR Ment Health. 2024 Jul 4;11:e52045. doi: 10.2196/52045 (PMC11258528; doi:10.2196/52045)
Supplement: Multimedia Appendix 1 [file mental_v11i1e52045_app1.docx]

## Multimedia Appendix 1

Table S1. Characteristics and prevalence of Depressive Symptomatology (DS) for the PROACTIVE dataset

|  |  | Number | % | Prevalence of DS (%) |
| --- | --- | --- | --- | --- |
|  |  |  |  |  |
| Gender |  |  |  |  |
|  | Female | 2400 | 63.5 | 35.1 |
|  | Male | 1380 | 36.5 | 22.4 |
| Education level |  |  |  |  |
|  | None | 573 | 15.2 | 34.9 |
|  | 1-4 years | 1789 | 47.3 | 33.0 |
|  | 5-8 years | 841 | 22.2 | 27.0 |
|  | >8 years | 577 | 15.3 | 23.4 |
| Minimum wage ^a^ |  |  |  |  |
|  | <R$ 998 | 2457 | 65.0 | 34.6 |
|  | R$ 998-1996 | 812 | 21.5 | 25.7 |
|  | R$ 1996-2994 | 301 | 8.0 | 17.6 |
|  | > R$ 2994 | 210 | 5.5 | 19.5 |
| Diabetes |  |  |  |  |
|  | No | 2524 | 66.8 | 27.2 |
|  | Yes | 1256 | 33.2 | 37.1 |
| Hypertension |  |  |  |  |
|  | No | 1311 | 34.7 | 23.3 |
|  | Yes | 2469 | 65.3 | 34.3 |

^a^ Brazilian Reais

Table S2. Characteristics and prevalence of Depressive Symptomatology (DS) for the PNS 2013 dataset

|  |  | Number | % | Prevalence of DS (%) |
| --- | --- | --- | --- | --- |
|  |  |  |  |  |
| Gender |  |  |  |  |
|  | Female | 26199 | 59.4 | 10.3 |
|  | Male | 17876 | 40.6 | 4.7 |
| Education level |  |  |  |  |
|  | None | 1102 | 2.5 | 12.1 |
|  | 1-4 years | 5762 | 13.1 | 10.1 |
|  | 5-8 years | 12583 | 28.5 | 9.1 |
|  | >8 years | 24628 | 55.9 | 6.8 |
| Minimum wage ^a^ |  |  |  |  |
|  | <R$ 678 | 19978 | 45.3 | 9.5 |
|  | R$ 678-1356 | 13441 | 30.5 | 7.7 |
|  | R$ 1356-2034 | 4275 | 9.7 | 5.9 |
|  | >R$ 2034 | 6381 | 14.5 | 5.3 |
| Diabetes |  |  |  |  |
|  | No | 40944 | 92.9 | 7.5 |
|  | Yes | 3131 | 7.1 | 14.5 |
| Hypertension |  |  |  |  |
|  | No | 33362 | 75.7 | 7.5 |
|  | Yes | 10713 | 24.3 | 14.5 |
|  |  |  |  |  |

^a^ Brazilian Reais

Table S3. Characteristics and prevalence of Depressive Symptomatology (DS) for the PNS 2019 dataset

|  |  | Number | % | Prevalence of DS (%) |
| --- | --- | --- | --- | --- |
|  |  |  |  |  |
| Gender |  |  |  |  |
|  | Female | 38672 | 54.5 | 13.9 |
|  | Male | 32235 | 45.5 | 5.8 |
| Education level |  |  |  |  |
|  | None | 2256 | 3.2 | 11.7 |
|  | 1-4 years | 9518 | 13.4 | 11.1 |
|  | 5-8 years | 19842 | 28.0 | 11.1 |
|  | >8 years | 39291 | 55.4 | 9.4 |
| Income ^a^ |  |  |  |  |
|  | <R$ 998 | 37057 | 52.2 | 11.7 |
|  | R$ 998-1996 | 18322 | 25.8 | 9.2 |
|  | R$ 1996-2994 | 6366 | 9.1 | 8.6 |
|  | >R$ 2994 | 9162 | 12.9 | 7.0 |
| Diabetes |  |  |  |  |
|  | No | 64617 | 91.1 | 9.6 |
|  | Yes | 6290 | 8.9 | 16.2 |
| Hypertension |  |  |  |  |
|  | No | 50670 | 71.5 | 9.6 |
|  | Yes | 20237 | 28.5 | 16.2 |

^a^ Brazilian Reais

Table S4. Features in the Bayesian model based on the PROACTIVE dataset

| **Name** | **Description** | **Response Value** |
| --- | --- | --- |
|  |  |  |
| AGE | Age | [60-69, 70-79, ≥80] |
| EDUCATION | Formal education level | [None, 1-4 , 5-8, >8 years] |
| GENDER | Gender | [Male, Female] |
| INCOME^a^ | Personal income | [<R$ 998, R$ 998-1996, R$ 1996-994, >R$2994 |
| LANDLINE | Do you have a Landline? | [Yes, No] |
| MOBILEPHONE | Do you have a Mobile phone? | [Yes, No] |
| FACEBOOK | Do you use Facebook? | [Yes, No] |
| FEELAGE | In general (or most of the time), how old do you feel? | [<50, 51-60, 61-70, >70 years] |
| BALANCE | Do you have problems with postural balance? | [Yes, No] |
| OUTOFBREATH | Have you ever experienced shortness of breath while walking, climbing stairs, or with changes in temperature (for example, when it's hot or cold)? | [Yes, No] |
| DIABETES | Have you been diagnosed with diabetes? | [Yes, No] |
| HYPERTENSION | Have you been diagnosed with hypertension? | [Yes, No] |

^a^ Brazilian Reais

Table S5. Features in the Bayesian model based on the PNS 2013 dataset

| **Name** | **Description** | **Response Value** |
| --- | --- | --- |
|  |  |  |
| N004 | Do you feel chest pain or chest discomfort when walking on a hill, go up one flight of stairs, fast walking? | [Yes, No] |
| N005 | Do you feel chest pain or chest discomfort when walking in a normal pace? | [Yes, No] |
| N003 | What is the difficulty level in general when it comes to moving? | [None, mild, medium, intense, can't move] |
| N002 | Do you use any aids such as a cane, crutches, wheelchair, walker, or other equipment to assist with mobility? | [Yes, No] |
| G006 | Do you have any physical disability? | [Yes, No] |
| Q084 | Do you have any chronic spinal problems such as chronic back or neck pain, lumbago, sciatica, vertebral or disc problems? | [Yes, No] |
| Q079 | Have you been diagnosed with arthritis or rheumatism? | [Yes, No] |
| Q132 | In the past 2 weeks, have you used any medication to help you sleep? | [Yes, No] |
| J002 | In the past two weeks, have you been unable to perform any of your usual activities (such as work, school, playing, household chores, etc.) due to health reasons? | [Yes, No] |
| J001 | In general, how is your health? | [Very good, good, normal, bad, very bad] |
| N001 | In general how do you evaluate your health? | [Very good, good, normal, bad, very bad] |
| J007 | Have you been diagnosed with chronic disease (mental or physical) for more than six months? | [Yes,No] |
| Q128 | Have you been diagnosed with another chronic disease (mental or physical) for more than six months? | [Yes,No] |
| Q030 | Have you been diagnosed with diabetes? | [Yes, only during pregnancy, no] |
| Q002 | Have you been diagnosed with hypertension? | [Yes, only during pregnancy, no] |
| Q029 | When was the last time you had a blood test to measure your blood sugar level? | [Within 6, 7-12, 13-24, 25-36, >36 months, never] |
| Q001 | When was the last time you had your blood pressure measured? | [Within 6, 7-12, 13-24, 25-36, >36 months, never] |
| J011 | When did you last consult a doctor? | [Last 12, 13-24, 25-36, >36 months, never] |
| X001 | When was the last time you consulted a doctor? | [Last 14, 15-30, 31-90, 91-360, >360 days, never] |

Table S6. Features in the Bayesian model based on the PNS 2019 dataset

| **Name** | **Description** | **Response Value** |
| --- | --- | --- |
|  |  |  |
| N004 | Do you feel chest pain or chest discomfort when walking on a hill, go up one flight of stairs, fast walking? | [Yes, No] |
| N005 | Do you feel chest pain or chest discomfort when walking in a normal pace? | [Yes, No] |
| Q06306 | Have you been diagnosed with heart disease? | [Yes, No] |
| Q068 | Have you been diagnosed with stroke? | [Yes, No] |
| J037 | In the past 12 months, have you been hospitalized for than 24h? | [Yes, No] |
| J046 | In the past 12 months, have you been in ER? | [Yes, No] |
| J002 | In the past two weeks, didn't do any daily activities because of a health problem? | [Yes, No] |
| J014 | In the past 12 weeks, have you looked for health professional? | [Yes, No] |
| H001 | When did you last consult a doctor? | [<15, 16-30, 31-180, 181-360, >360 days] |
| J01101 | When was the last time you consulted a doctor? | [<1, 1-2, 2-3, >3 years, never] |
| J007 | Have you been diagnosed with chronic disease (mental or physical) for more than six months? | [Yes, No] |
| Q128 | Have you been diagnosed with another chronic disease (mental or physical) for more than six months? | [Yes, No] |
| Q074 | Have you been diagnosed with asthma? | [Yes, No] |
| Q11604 | Have you been diagnosed with another chronic lung disease? | [Yes, No] |
| Q00201 | Have you been diagnosed with hypertension? | [Yes, No] |
| Q00101 | When was the last time you had your blood pressure measured? | [Within 6, 7-12, 13-24, 25-36, >36 months, never] |
| Q02901 | When was the last time you had a blood test to measure your blood sugar level? | [Within 6, 7-12, 13-24, 25-36, >36 months, never] |
| Q05901 | When was the last time you had a blood test to measure your cholesterol? | [Within 6, 7-12, 13-24, 25-36, >36 months, never] |
| Q132 | In the past two weeks, have you used any medication to help you sleep? | [Yes, No] |
| V00202 | In the past 12 months, has anyone yelled or cursed you? | [Yes, No] |
| V00203 | In the past 12 months, has anyone used social media to attack you or expose photos without your consent? | [Yes, No] |
| V00201 | In the past 12 months, has anyone offended you in front other people? | [Yes, No] |
| V00205 | In the past 12 months, has anyone destroyed something from you on purpose? | [Yes, No] |
| V00204 | In the past 12 months, has anyone threatened to hurt someone important? | [Yes, No] |
| V01402 | In the past 12 months, has anyone pushed you? | [Yes, No] |
| V01405 | In the past 12 months, has anyone threatened with a knife or guns? | [Yes, No] |
| V01401 | In the past 12 months, has anyone slapped you? | [Yes, No] |
| V01403 | In the past 12 months, has anyone punched or pulled your hair? | [Yes, No] |
| V01404 | In the past 12 months, has anyone strangled or burned you on purpose? | [Yes, No] |


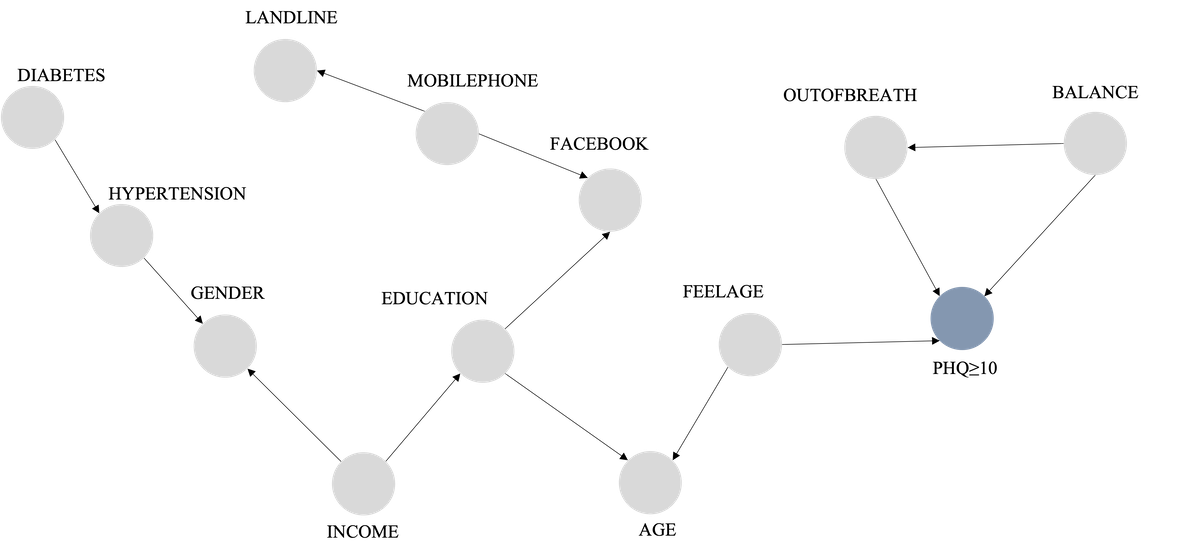


Figure S1. Bayesian network for PROACTIVE


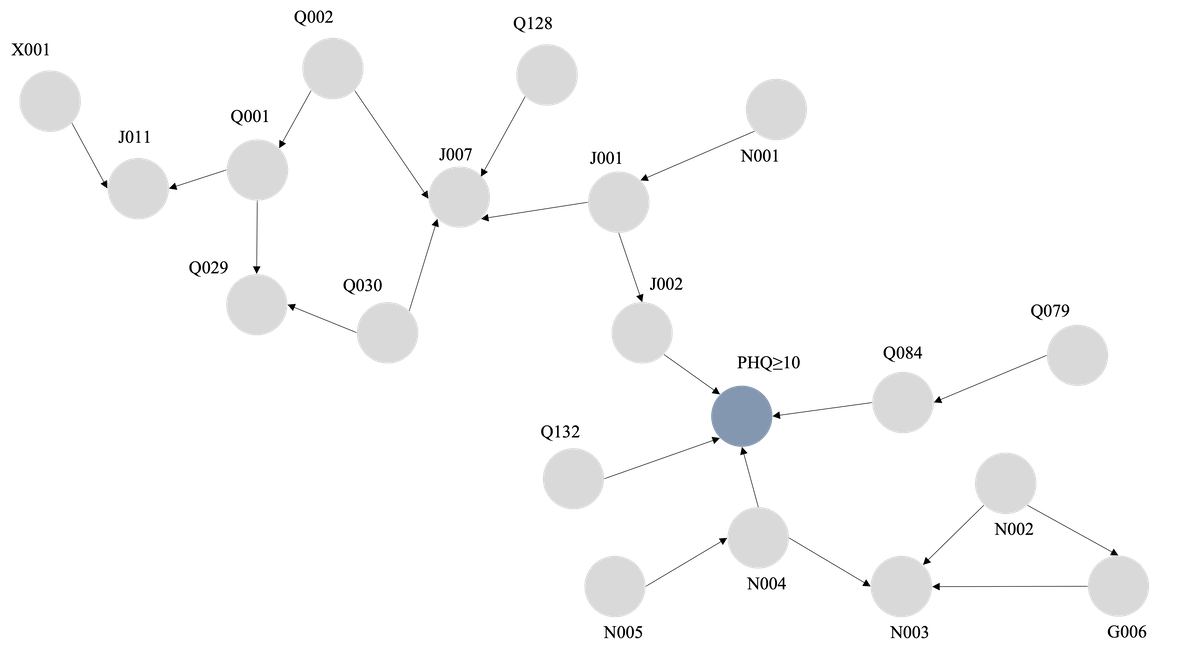


Figure S2. Bayesian network for PNS 2013


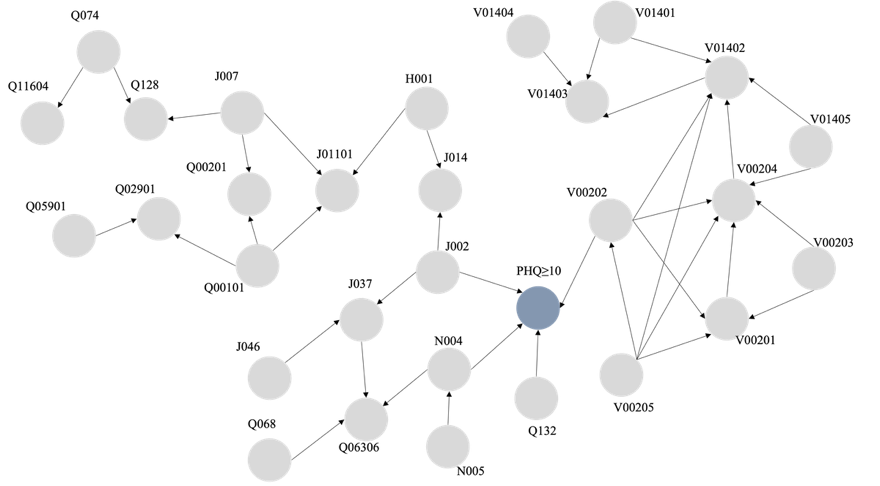


Figure S3. Bayesian network for PNS 2019
